# An Interview with George Whitesides

**DOI:** 10.1089/rorep.2023.29007.intgw

**Published:** 2023-12-11

**Authors:** Marwa ElDiwiny

**Affiliations:** Marwa ElDiwiny, Vrije Universiteit Brussel, Brussels, Belgium. George Whitesides, Department of Chemistry and Chemical Biology, Harvard University, Cambridge, Massachusetts, USA.

**Figure:**
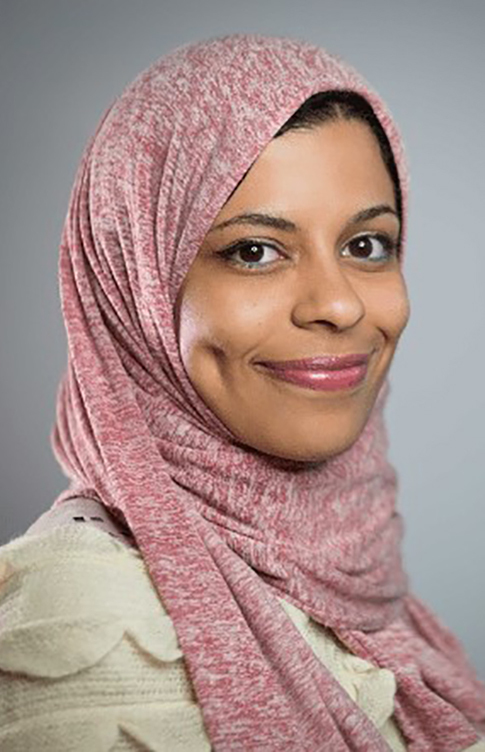
Marwa ElDiwiny

**Figure:**
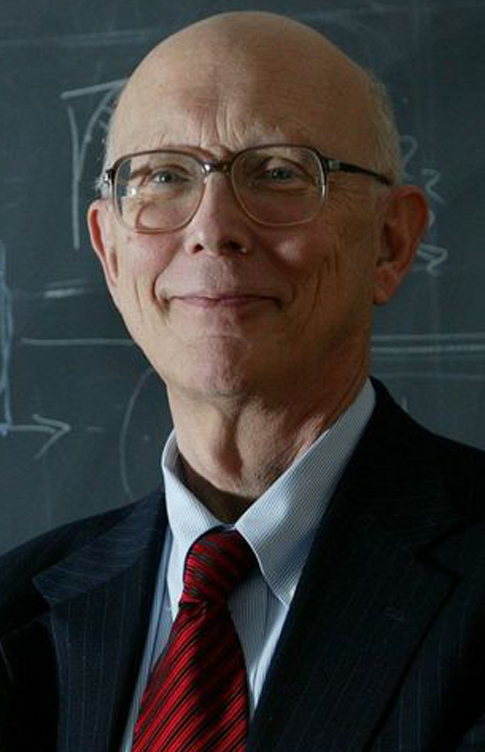
George Whitesides

***Marwa ElDiwiny:* How do you see the soft robotics field so far? Are you satisfied with the progress?**

**George Whitesides:** The progress in soft robotics is going well. There are many people working on it, coming up with new things, so it hasn't finished the first stage of adventure.

***Marwa ElDiwiny:* Do you think there are missing pieces we still didn't really consider in the research before?**

**George Whitesides:** It's not, I think, so much a question of research. The question is of finding initial demonstrations that will stimulate good companies with good engineers to think about taking the next step, because what you can do at university is to invent first prototypes of new ideas. What a real application requires is that someone think through the engineering. There isn't the kind of synergy that occurs where you have a number of companies thinking in computing around a similar technology.

***Marwa ElDiwiny:* Do you think we should be more focused on the benefit of simplicity in design and also cost wise?**

**George Whitesides:** I think a lot more effort can always go into that step between university and industry, and when one is talking about a new technology, the objective is to get good industrial people to think about it as soon as they can. That's beginning to happen. What one wants is more companies and more engineers thinking about the parts that actually work on the shop floor.

It's always the case when you have working technology in someone's factory that someone else can look at that and say, “Oh that's really working well. I could modify it,” and the modified version would then help me to solve problems that I have. At present, we don't have quite enough people producing chemically and industrially relevant technology as opposed to chemically and real-world relevant technology. Transition is occurring. I don't think it's necessarily slow but it's slow in every field where it finally occurs. It took a long while for the transistor to happen.

***Marwa ElDiwiny:* What makes a good soft robotic design for real-world application?**

**George Whitesides:** I don't know about soft robotic design, but I think for new technologies it takes 10 years and $10 million, and those are very approximate numbers. The point is that it's a hard and slow step to get something to the point to become routine for the users as opposed to routine for specialists who do only that in an academic laboratory, and in which if something doesn't work, it's not the end of the world.

The one company that I have contact with is Soft Robotics Inc., a local company that has found an application that is in food handling. It's done very well with this and it's now becoming a reality. The particular advantage there is that you could do things fast, it's not as expensive as other ways of doing it, and you get people out of messy jobs. You require a soft touch because fluid is soft. Soft robotics is one, but it would be nice to have a couple of others working in different areas to come up with new ideas.

***Marwa ElDiwiny:* What kind of new use cases might make a good case in industry now beside the one example of Soft Robotics, Inc.? What other use cases do you think may be interesting now in the market?**

**George Whitesides:** We're going to work on applications in biomedicine, particularly replacing the tasks that nurses do that are repetitive and require skill. Some of that can be done I think with robots. Robots are very reproducible. I don't know whether that could be made to work. We've made one demonstration of a technology for making a cast for children with broken bones on the arm. This works for an odd reason, and that is the kids don't like having a saw remove the plaster cast when they're finished. It's complicated for the kid.

If that can be made to work, then there's an entry point and one can begin to make other analogues for larger limbs and for adults and for different kinds of problems. The underlying issue of how you make the problem from a laboratory demonstration to a commercial reality is one of the things that we in the United States have to learn how to do better. We do this, I think, pretty well in the electronics world, but there are many places where we don't see it.

If you look at the length of time that was required to get 3D printing into commercial production and new kinds of polymers into commercial production. That all took a long time. How do we do that more rapidly? What do you need to do before people begin to use a new technology to solve real problems and to think of a new technology to solve real problems. I'm deeply interested in that question. Soft robotics is an example of a technology for doing that kind of thing.

***Marwa ElDiwiny:* Can you tell me how you can think about combining low cost and still maintaining functionality in the software robotic context?**

**George Whitesides:** Soft robotics tests to do something that works well at the level of cost in addition to working well at the level of just being able to perform some function. This is, again, something that universities typically don't do very well. There are places that do but, in general, it's not straightforward to do that. What we do is invent new things and make them available for industry, if industry would like to take them on, and then try to apply them to a definite objective and use. The question is, “What can the university do to make this grow more rapidly?” One thing is to come up with new technologies and new ideas, but there may be other things as well, and that problem hasn't been solved yet.

***Marwa ElDiwiny:* Where do you think the solution could be?**

**George Whitesides:** In general, where is the soft touch required? Nursing is an example. Working with soft robotics for food is an example because food requires soft touch. Where you see ideas that require soft touches, then you can apply interesting applications. Adam Stokes and some others in the United Kingdom are working on undersea applications and soft robots work very well undersea, because living in a salty aqueous environment doesn't bother the polymer at all. That's an area where there's an enormous amount of technology that's involved in undersea pipelines and in observing cables and in things of that sort. That's the right solution for a problem. I'm not at all discouraged by the rate at which the field is developing. But is it a mature field at which no more invention is necessary? The answer is no. More invention is necessary.

***Marwa ElDiwiny:* Do you think sometimes we use soft robotics for the wrong solution?**

**George Whitesides:** A lot of what goes on in the university is to demonstrate that something can be done. Whether that demonstration of what can be done, assuming it's successful, corresponds to a real problem is a different story altogether. Typically it's a problem that a company can handle better than a university, because they could do the cost economics, they'd build on the basis of performance, and they look at issues like what's the uptime and the downtime of this new technology. Then we provide a box that you open up and there's something inside already in your assembly line and your packaging line that you're ready to go. That's impressive. I think things are going well, but there's still more to be done.

***Marwa ElDiwiny:* I want to ask you about the actuation in soft robotics compared with what we see in the quadruped robot, for example. Do you think we have to develop or invent artificial muscle that resembles the biological muscle that could have the same strengths of quadruped but is still soft? Do you think we can merge the two?**

**George Whitesides:** The advantages of having a technical feature you've made up, so there's nothing that says my muscle is the right strength and the right size to go down the pipe and find an explosive charge at the end that needs to be destroyed. The soft robot can do that, because if it is the right strength to do it and then is destroyed in the process, well, that's too bad but it's not the end of the world, because that may well be what is designed to be done.

The solution is to have a range of operating principles and a range of materials and a range of types of evacuation. One of the areas that we've been working on within the last period of time is having soft robots that also act as digital devices just by themselves. That is the system, instead of working on electrical current, works on pressure and fluid movement.

You may ask whether that makes sense. The answer is that if you look at Feynman's book on computation, the analogies that he uses are mostly for electronic devices and fluidic devices. Yes, I think it can be made to work. Will that be the answer to a problem? We'll see.

If you look at the length of time that's required for any new technology and the rate of development, a good comparison is microelectronic devices, computer chips, and then DNA sequencing, because the basic ideas were established at about the same time, in the late 1950s. It's taken a much longer time for DNA to get up to speed, but DNA sequencing is now working incredibly well. It's working much better than I think I would've ever expected, so be patient. In 10 years, you're going to ask the same questions again and ask how much change there's been.

***Marwa ElDiwiny:* Do you think we have a deep understanding of the morphology and architecture in soft material, the soft structures that can give us emerging behavior or interesting behavior versus using control or artificial intelligence (AI)?**

**George Whitesides:** The answer is no. This is a good place for further research, because one community that's very relevant to the subject is the polymer or material science community. Can we make materials that are like polydimethylsiloxane (PDMS) but resistant to fire? Can we make things that are opaque to transmission of gases or things? There are all kinds of polymers for all kinds of purposes. The question is, “Can you make a variety of different functions or designs in robotics based on new kinds of materials?” The answer to that is yes. We use a number of different polymers but we're really not a good polymer scientist. That's not our strength.

If you look more broadly at the field of science or engineering and you bring in the best of digital engineering and then material science and what kinds of materials can be molded and what are they good for and how much do they cost, you come up with new answers. That hasn't yet happened. It's a bridge that's been difficult to cross over because there aren't that many people who know about materials, who also know about engineering devices.

We have the solutions for all sorts of problems. We have ways of making, for example, PDMS robots less combustible. But having said that, do we have a clear application where that's a good thing to do? One of the interesting things about this area is that the conventional robots community pretty strongly resists this area when it comes to peer-reviewed funding. It's a little bit of a struggle still to come up with good ways of doing things when people basically say, “This isn't the right way to do it.” I'm not too concerned, but it is something that needs to be dealt with.

***Marwa ElDiwiny:* What do you think about publish or perish?**

**George Whitesides:** I'm not concerned with this, I just think it's a little bit of a problem the field has to overcome. The practicalities in the university are that, if you don't put your work out there, you don't get more money to do more work with, so you've got to publish. You can come up with one good article that really opens doors and science in some ways and then there's all kinds of things you can do with it and others can do with it. I do think that established communities tend to protect their own supply of money, whatever that turns out to be. Is it, in general, a good idea to publish just to publish and get more names on a publication record? Probably, the answer is no. But unless what you're doing is moving the field in some direction forward, probably that's not a good idea.

***Marwa ElDiwiny:* How do you find a good problem?**

**George Whitesides:** There are problems in which the users want a better solution and sometimes those are problems for which you can provide a better solution with development and sometimes you can't. You just can't figure out how to do it.

For example, we've had the problem in health care of cancer for as long as I've known anything about this field. And there's been progress. Sometimes really good progress, but it remains a problem to solve for now. Is there something out there that makes this new and interesting that we can approach the problem in a new way or not? Or is it simply we're stuck with what we've got? And I think the answer is going to be some of both. That there'll be cases where we've got an idea that's the best idea that's around, and other cases in which we haven't yet tried anything new.

The same thing for robotics. Does one really need a robot to walk on the inside of an oil pipeline and inspect for leaks? What does one need? Something that can go undersea and look at an entry hole for a pipe and find out whether it's oozing oil around the sides. The answer is, I don't know. I'm not a drilling engineer. I don't know whether there's a problem there, and if there is a problem, would you solve it using a soft robot?

There will be certainly cases where I can think that soft robotics is the right way of doing it, and other cases where I don't know. I know that soft robotics brings soft touch, and I know that soft robotics are relatively inexpensive to make relative to many hard robots that are able to do the same kinds of things or similar kinds of things. There will be different answers depending upon the circumstances and there's probably a room for both since robotics is a field that's going to expand over the next period of time simply because there's more need to generate solutions to problems than there are people to generate those solutions using existing technology.

***Marwa ElDiwiny:* What is your take about the robot we have now from Boston Dynamics and other companies, and for example, from the humanoid robot? What do you think about these technologies that are very expensive, but are getting attention from the market?**

**George Whitesides:** I think about all of the considerations, such as: Does it work? How much does it cost? What is the cost of operation over a period of time? Are there other problems that come into it in terms of toxicity or that determine what makes a good product? We had chips and we had vacuum tubes, both of which solve problems, actually similar problems. It's turned out over the course of time that transistors work much better than vacuum tubes, and so the world has gone that way.

What is needed is to figure out how to make the best vacuum tubes, the best transistors, or the best soft robots of a variety of sorts, or the best hard robots of a variety of sorts and then see which robots actually work best. A lot of science and engineering is empirical. What works best? One or another can work best at different times or with different materials or with different methods of construction or powering. These are all good, legitimate questions.

***Marwa ElDiwiny:* How do we begin to envision where the field of robotics will go?**

**George Whitesides:** If you think about electronics, nobody thought at the beginning that they would make a transistor, and that the transistor would then lead to the World Wide Web. You just didn't think about that. It came because pieces were added, and functionality was added. The field developed based on the pieces that were available to make it develop. Robotics is also a very big area. I think that with the advent of AI, it may turn out to be, in some ways, a bigger area. Instead of shipping information around, what you're doing is replacing people in mundane jobs with machines and allowing people to do more. We don't know whether that particular replacement is going to happen as indicated or whether it's going to be something less benign.

What we're going to do is try it and we will see what happens when we've had 20 years of experience with AI and with soft robotics and with hard robotics and with the web and communication transfers and all the rest of this. It's going to be a different place. I am certainly not going to see the end of this. I think that even you, though you're young, are not going to see the same kind of experience. Or you're going to see the complete experience and it's going to be just immensely interesting to see it develop.

With the problem of communication and calculation, we see a variety of options going after new problems. And with things that require touching and moving and drilling and doing other things with mechanical objects and mechanical tasks, we haven't seen the same explosion of ideas. I think that soft robotics will be one of those but, they'll probably be others as well, different forms of hard robotics.

What hard robots are doing now in terms of dexterity and touching and feeling as they touch are all very interesting. Many of them can be used as soft robots as well. You can already see a combination of hard and soft robots; the primary industrial uses of soft robots are as grippers that you put on the end of existing hard robotic arms. The arms provide the speed and the strength and the soft grippers provide the ability to handle the things that you're working with and that a combination works very well.

***Marwa ElDiwiny:* Will we see more combinations of soft and rigid robots?**

**George Whitesides:** There's going to be things that are purely soft and things that are purely hard and things that are in between. The point, which is the interesting point that people bring up repeatedly, is if you are successful in a new technology, it tends to disappoint people.

To me, one of the most interesting examples of a benign use of a soft robot is in washing machines and dishwashers. They took a very large class of humans, otherwise known as women, and relieved them of the task of washing dirty dishes and dirty underwear and allowed them to do other things like being CEOs. What robots of all sorts can do is to relieve people of repetitive, not-so-interesting tasks and enable them to do less repetitive, more interesting tasks. And I'm an optimist in thinking that's the way the field should and will develop.

***Marwa ElDiwiny:* Do you think it's a political decision when it comes to the cost of accessibility or just for the health care sector?**

**George Whitesides:** Everything is a political decision. If you have a new idea, it almost always displaces an idea that already exists that isn't working well enough. People don't usually see any reason to replace working technology with new technology, because new technology always comes with problems. The easiest cases are the ones in which there are no existing solutions. Even when there is an existing solution, you need to ask, “Is something new needed here? And if so, why is it needed and what is it needed for and how much is it going to cost?” An interesting example in soft robotics is AI.

When people were setting up the first electrical systems, there was the basic question of how you ship power over long distances. Do you do AC or do you do DC? There were arguments for both. In soft robotics, though, the question is whether it provides soft touch, nonlinear responses to stimuli. Does anyone know? The answer is, in some cases, yes. In some cases, not yet. We'll have to see.

***Marwa ElDiwiny:* Is pneumatic actuation a practical solution?**

**George Whitesides:** I like to do it at a distance. I have a pump in one room and I have a device that is doing something, and all I have connecting them is a thin tube. It's not that much different than electrical activation, except that I don't happen to have an electric motor with all its weight and expense and all the rest of it on one side and the other side. I don't happen to have a dynamo. I think that pneumatic actuation is a very good way of providing fine control at different points in an operation cycle. If you think about where pneumatic actuation works, think, for example, about automobile tires. It's very difficult to think of some of the methods of actuating a flexible piece of rubber so it becomes stiff and hard enough but not too hard.

Another thing that's good about pneumatic actuation is that it's very lightweight, and it's also very cheap. In principle, you can develop methods of doing electrical actuation that don't involve having big pumps and things of that sort, then tubing and some source of acid or base or whatever it might be.

***Marwa ElDiwiny:* Why do you think academicians tend to be complex?**

**George Whitesides:** What one always looks for in science, curiosity leads you to ask, “Here's a system that's important, and it has lots of different moving pieces—complexity and blood flow and whatever it might be. Can I understand it?”

The answer is that one way of understanding anything is to build other systems that have some of the same characteristics. Nobody has yet put together a really good theory of complexity. We have people who've made very good progress looking at complex systems in answering questions about them. But can we say, like in quantum mechanics, that we really understand these things, even if we don't understand them, can we model them? The answer is no, we can't. In my opinion, complex systems are good ones to look at, and I would say are areas of science that are probably most interesting.

***Marwa ElDiwiny:* Have you had any moment of doubt in what you've been doing? Has any experience in your life changed you?**

**George Whitesides:** You don't have to worry about doubt when you solve the problem, but if you solve the problem, you stop thinking about it anyway. It's no longer fun to think about. What I do as a scientist is I think about complex problems and then try to understand how you reduce them to things that are less complex, then try to understand the pieces. Look for ways of making all of that as simple as possible so that you end up with things that are predictable, in which you can understand what the response of your system is going to be to a given change in its environment, changing its stimulus.

If I'm looking at a flame, how do I put out a fire without disturbing whatever the fire is burning? We don't know how to do that right now, in general. Take almost anything and pour water on it and you do more harm than the fire was doing in the first place.

Taking the example of electromagnetism. When we first started out, yes, it's true that we could run current through a wire and generate magnetic field or spin a rotor and get that to generate a magnetic field or electrical field, depending on what you're going to do.

The fact of the matter is that it took a long while to go from there to our modern electric-based society. It took people being interested in parts of this little complex array of phenomena that go under electromagnetism. It's all interesting, but what distinguishes the parts that are worthwhile working on. Some parts are not the answer, but some parts solve problems. Solving problems is a good stimulus toward simplicity, and that's one of the things that science and engineering should do.

***Marwa ElDiwiny:* How do you keep yourself young in your mind?**

**George Whitesides:** To me, much of the motivation in science is curiosity. There are an almost infinite number of things in the world that are interesting, and some of them are very important. The example of something that I'm interested in now and can understand is magnetism. If I say to you a current running through a straight upper wire generates a magnetic field, you may say, “Ho-hum.”

If I ask why does it do that, I think you'd have to struggle a bit to come up with a solution that I could understand. If you present me with Maxwell's equations, I would state to you that Maxwell's equations are one side of the phenomenon. They're very descriptive, but they don't explain at all why it happens. I think that the world is full of an almost infinite number of neat things that happen for reasons that we don't understand at all. Human beings, of course, are among the very neat things that happen that we don't understand at all or very well.

***Marwa ElDiwiny:* Does it bother you if you don't know the truth of something?**

**George Whitesides:** Let's take an example. One of the things we are working out right now is the origin of life. You can think of a number of ways of which this might happen, and you can demonstrate some parts of some of those processes in the laboratory and other parts of them you can't. People are coming up with really good and interesting solutions, which may or may not have anything to do with the origin of life, but certainly generate new avenues of research.

Does it bother me that we can't even solve the problem yet? Yes, it does. I would like to think that having spent many years studying this, we would know more about it than we do. One alternative is the formation of the engineering of surfaces and self-assembled materials was one way of doing that kind of thing.

That has converted what was a very difficult area, which was making surfaces that had different properties, into an area in which it's easy to make surfaces with different and predictable properties that allow you to test all sorts of hypotheses.

Curiosity in that area combined with a certain amount of luck has generated a system that everyone can use to ask and answer the questions they have about these surfaces. Surface engineering or surface chemistry in the form of model layers has been very successful in the area of origin of life.

***Marwa ElDiwiny:* Yeah. Maybe a quick question here. Do you believe as an observer like… I don't know, I'm curious from the way you think. If you don't want to answer question, but I'm curious. Do you think there's a God or high power at just purpose for the life? I'm curious what you think. If you can share, okay. If not, I respect that.**

**George Whitesides:** I would say one thing about God, G-O-D, is if there is a conscious entity that can control a wide variety of things I can't even comprehend, I can never understand the mind of this entity, whatever it's going to be because I can't. And so I know myself. My job, after all, is to understand, be curious about things, to try to understand them, and then to try to use that understanding so that I can benefit my society and my profession and people around me. And that's a different question altogether.

***Marwa ElDiwiny:* Do you have any message or questions you would like to leave for the robotics community to think about?**

**George Whitesides:** I think the main thing that drives things forward is curiosity. That includes all sorts of questions about how birds fly and why insects are mostly hard on the outside and soft on the inside, for example.

Then there's the engineering question of how do you help your fellow human have a better life with what you come up with? There is a distinction between understanding to write articles and to solve problems to benefit society. Writing articles that go into professional journals is very important. My specification for a good life is to start with curiosity and keep in mind that the end product should benefit society. Good luck!

